# Genetic and Ecological Relationships of *Anastrepha ludens* (Diptera: Tephritidae) Populations in Southern Mexico

**DOI:** 10.3390/insects11110815

**Published:** 2020-11-19

**Authors:** Lorena Ruiz-Montoya, Rodrigo Verónica Vallejo, David Haymer, Pablo Liedo

**Affiliations:** 1 Departamento de Conservación de la Biodiversidad, El Colegio de la Frontera sur (ECOSUR), Carretera Panamericana y Periférico Sur s/n, San Cristóbal de las Casas, Chiapas 29290, Mexico; rodvv@hotmail.com; 2 Department of Cell and Molecular Biology, 1960 East-West Rd, Biomed T511, University of Hawaii, Honolulu, HI 96822, USA; dhaymer@hawaii.edu; 3 Departamento de Agricultura, Sociedad y Ambiente, El Colegio e la Frontera Sur (ECOSUR), Carretera Antiguo Aeropuerto km 2.5, Tapachula, Chiapas 30700, Mexico; pliedo@ecosur.mx

**Keywords:** area-wide pest management, enzymatic loci, insect-plant interaction, Mexican fruit fly, population genetics of insects

## Abstract

**Simple Summary:**

The Mexican fruit fly, *Anastrepha ludens*, causes extensive damage to important agricultural commodities wherever it is found. Any effort to control or limit the damage caused by this pest requires a thorough understanding of the genetic makeup of the populations found in a particular area. Our study focused on flies found in the Soconusco region of southern Mexico. Flies in this region are typically found infesting different types of fruit trees that are either cultivated or naturally occurring. For our study, we collected male and female flies from four different types of fruit trees in several specific localities in the Mexican state of Chiapas. We analyzed the genetic makeup of a total of 725 flies in an attempt to look for differences that might be associated with the sex of the flies, the specific plants they were found on, or specific localities within the study area. We found a lot of genetic differences in flies from the various collections, but these were not strongly associated with different types of fruit trees or the specific collection site. From this, we concluded that the populations of flies from this entire region were largely similar.

**Abstract:**

Knowledge of the influence of evolutionary factors that promote either the differentiation or cohesion of pest insect populations is critical for the improvement of control strategies. Here, we explore the extent to which genetic differentiation occurs between populations of the Mexican fruit fly, *Anastrepha ludens*, in association with four plant hosts (*Citrus sinensis*, *C. paradisi*, *Mangifera indica* and *Casimiroa edulis*) in the Soconusco region of Chiapas (Mexico). Using variants from six enzymatic loci, we obtained measures of genetic diversity for three sample arrangements: (1) by sex per locality, (2) by locality and (3) by host. The extent of genetic differentiation in populations was assessed using the Analyses of Molecular Variance (AMOVA) method for each array of samples, and moderate to high levels of genetic variation were observed between the sexes, as well as among localities and host plants. A Bayesian approach was then used to assess any population structure underlying the genetic data we obtained, but this analysis showed no significant structuring due to locality or host plant. We also considered whether the observed genotypic frequencies in male and females matched those expected under a hypothesis of random mating. Here we found significant deviations from expected genotypic frequencies, suggesting that sexual selection is acting on these populations. Overall, our results indicate that sexual selection, along with the presence of some heterogeneity in environments provided by both geographical factors and availability of host plants, has influenced the evolution of pest populations in this region of Mexico. Implications for area-wide pest management strategies are discussed.

## 1. Introduction

Both genetic and ecological factors can promote either the differentiation or cohesion of pest insect populations and understanding these phenomena has become increasingly important for the improvement of control strategies in specific areas. For example, Wright’s *F* statistics can be used to estimate levels of genetic differentiation within and between populations of pest insects [1], and this can be useful to decide on specific management strategies tailored to each locality or region [2].

In Mexico, *Anastrepha ludens* (Loew) (Diptera: Tephritidae), also known as the Mexican fruit fly, is one of the main pests of cultivated fruit trees, and this species has been the subject of several monitoring and control programs [2]. Currently, this species significantly impacts the production and marketing of several cultivated fruits, including oranges (*Citrus sinensis* L) and mangoes (*Mangifera indica* L.) [3,4] in Mexico as well other countries in Central America [5,6].

Plants of the family Rutaceae, such as *Casimiroa greggii* (S. Watson) F. Chiang and *Casimiroa edulis* Llave & Lex., have been recognized as the native hosts of *A. ludens* [7]. Orange and mango were introduced to Mexico during the time of Spanish colonization [4,8,9,10], and *A. ludens* has shifted to use them as hosts. In addition, plants from at least 22 species from taxonomically distant families have been reported as hosts for this pest [4]. This range of host plant usage may allow this pest to survive by moving from one host species to another, depending on their availability in specific localities [11].

The evolution of *A. ludens* has probably occurred in close relationship with these host plants, as has been proposed for other species of phytophagous insects [12,13,14]. The colonization of new host plants also promotes a process of population differentiation that can eventually lead to adaptive speciation [15,16]. This has been proposed for *Ragholetis pomonela* (Tephritidae) in relation to the local host species *Crataegus* ssp. (Rosaceae) and the introduced *Malus pumila* (Rosaceae); in this case, the genetic differentiation occurred in relation to asynchrony in the phenology of host plant species [17]. Such specialization to host plants can also be limited by the level of gene flow between populations associated with each host species. This specialization can include factors such as the force of selection that each plant exerts on the pest populations [18] and by the amount of the phenotypic plasticity of the genotypes [19,20].

Genetic exchange in populations of insects associated with specific host plants may also decrease if selection occurs on preferences or recognition of the host plant, and if selection overcomes the effects of gene flow [20]. For *A. ludens*, under laboratory conditions, studies have shown random mating and a general lack of preferences for specific host plants. However, according to Aluja et al. [21], the host plant or the geographic origin can affect specific parameters such as the time of copulation, and that this is more prolonged in individuals of a common geographic origin. It has also been observed that the larvae of *A. ludens* have a greater survival rate in mango as a host plant compared to that seen in orange or guava [22]. These results suggest that host plants do exert selection pressures that can impact the genetic structure of populations of this pest.

Previous studies of *A. ludens* have revealed that this species has high levels of genetic variation and population structuring consistent with the presence of four subpopulations. The subpopulations occur within a distribution range corresponding to: (1) Western Mexico, (2) Eastern Mexico/Texas, (3) Guatemala/Belize/Honduras and (4) Costa Rica/Panama [6]. The Mexican populations in particular have been shown to exhibit moderate population structuring at a broad geographic scale [23], along with a stronger structuring at a narrower geographic scale [24,25]. However, studies exploring the influence of hosts on the genetic structure of *A. ludens* from Veracruz, Tamaulipas and Nuevo Léon (Mexico) concluded that they did not find that the structuring was attributable to either the geographic origin or the host [26], as had been suggested for other *Anasptrepha* species, specifically *A. fraterculus* [27] and *A. suspensa* [28]. In *A. fraterculus,* there appears to be an incipient speciation process promoted by factors such as environmental differences, host species preferences and their distribution [29,30,31].

Studies of sex specific genetic variation in *A. ludens* have also been lacking, even though genetic differentiation between the sexes is likely given its mating behavior or due to differential selection between the sexes [32,33,34]. In *A. ludens*, matings are known to occur in lek arenas where groups of males gather on plant substrates to display. Females are attracted to these mating leks, and it is here that sexual selection for mate choice takes place [35]. Non-random female choice for mates may involve preferences for specific male genotypes or phenotypes that may in turn promote changes in gene frequencies between the sexes. In this way, female mating success could explain much of the genetic variation seen within populations. Also independent of mating behavior, females and males may also have different genotypic frequencies because of differential selection or patterns of gene flow between populations [33].

Our objective here was to look at possible factors affecting the genetic variation and population structure of *A. ludens* in the Soconusco region of Chiapas, Mexico. Our first aim was to determine the effect of host plants. A second aim was to look at the possible effect of environmental conditions in different localities, and a third aim was to explore whether there is sex specific genetic variation that might affect the structure of populations.

## 2. Materials and Methods

### 2.1. Study Area and Environmental Factors

All localities are within a region with environmental characteristics as follows. The Soconusco Region in Chiapas, Mexico, is located at 15°19’ N latitude and 92°44’ W longitude in the so-called Coastal Plain of Chiapas and Guatemala. Elevation data is available per each locality, and overall this ranges from 0 to 4030 m above sea level at the summit of the Tacaná volcano. The predominant climatic conditions are sub-humid and humid–warm with summer rains. During the months of May to October, the average minimum temperature ranges from 21 °C to 22.5 °C, and the maximum from 33 °C to 34.5 °C. During this same period, rainfall ranges from 1200 to more than 3000 mm. In the period from November to April, the average minimum temperature ranges from 18° C to 19.5° C and the maximum goes from 32 °C to 33 °C. Rainfall during this period ranges from 75 mm to 800 mm. Land use is mainly for agriculture (48.76%) and cultivated grassland (26.64%) [36].

### 2.2. Collection of Anastrepha Ludens Specimens and Host Plant Species

**Figure 1:**
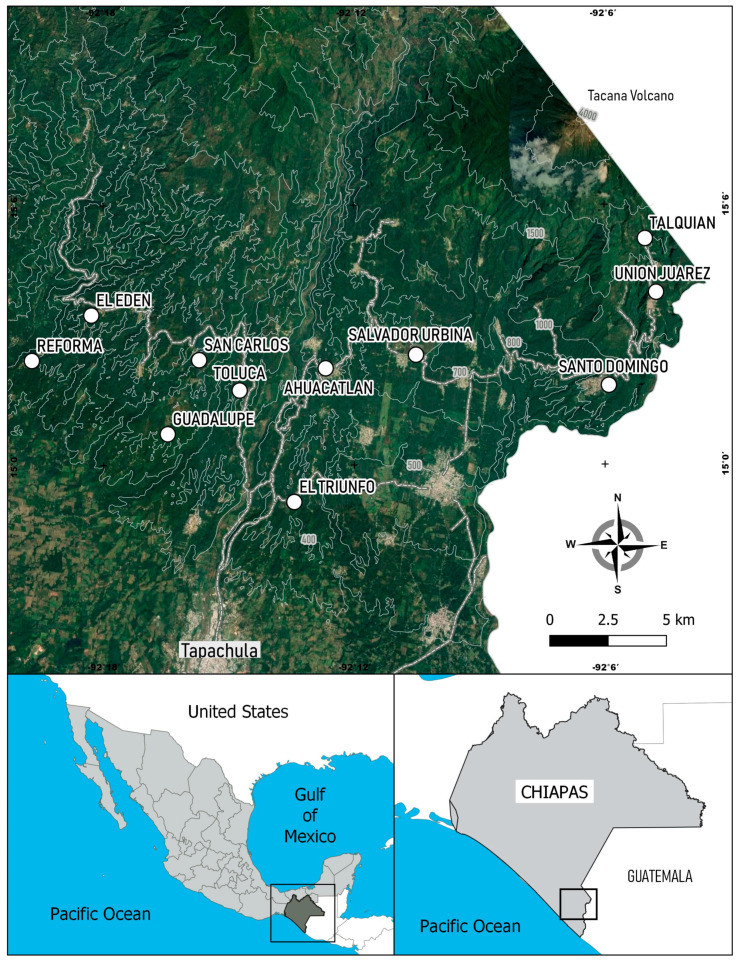
Map of sampling sites of *Anastrepha ludens* in Soconusco, Chiapas, Mexico.

Localities were identified in the Soconusco region where recurrent incidences of *A. ludens* have been recorded in the host plant species *C. sinensis* (CC), *M. indica* (MI), *C. paradisi* (CP) and *C. edulis* (CE) [5,37]. Male and female samples of *A. ludens* associated with these different plant species in various localities were collected for analysis. Three of these plants, *C. sinensis*, *C. paradisi*, and *M. indica,* are introduced species grown commercially. In addition, plants of one native species, *C. edulis,* were also used. The initial collection effort was aimed at locations where at least two of these host species were present, but this was not feasible. In the end, collections were made at 11 locations, and two of the host species were present in three of these sites (Table 1, Figure 1). The collections were made from January to March. Three fruits showing the presence of fly larvae were collected from each tree and at least four trees of each host species. The fruits were transferred to the laboratory, where third instar fly larvae were recovered. The larvae were placed in containers with vermiculite to induce pupation and later to obtain adults. Once the adults emerged, they were confirmed to be *A. ludens*, separated by sex and were stored at −70 °C in liquid nitrogen until genetic analysis.

### 2.3. Genotyping

We assayed 15 enzyme loci following the procedure described by Hebert and Beaton [38]. Of these, only eight showed reproducibility with clear and consistent activity. The genotype of each individual was obtained based on the pattern of bands of these eight enzymes, as revealed by cellulose acetate electrophoresis. The runs were carried out under ambient temperature at 55 V and 30 mA for 90 min in a CAMP [Citric acid, 4-(3-aminopropyl) morpholine] buffer solution, following the protocols of Herbert and Beaton [38]. The eight enzyme loci analyzed were: malate dehydrogenase (1.1.1.37, MDH), malate dehydrogenase NADP (1.1.1.40, ME), isocitrate dehydrogenase (1.1.1.42, IDH), 6-phosphoglucanate dehydrogenase (1.1.1.44, 6PGDH), glucose-6-phosphate dehydrogenase (1.1.1.49, G6PDH), aspartate amino transferase (2.6.1.1, GOT), glucose-6-phosphate isomerase (5.3.1.9, GPI), and phosphoglucomutase (5.4.2.2, PGM). Loci and alleles were recognized through observation of the staining pattern of each enzyme and following the recommendations of Hebert and Beaton [38] for the assignment of genotypes and loci. The GPI and MDH loci were not included in the analysis because they were detected as monomorphic.

### 2.4. Population Genetic Analysis

#### 2.4.1. Genetic Diversity, Male and Female Hardy–Weinberg Equilibrium Tests and Tests for Random Mating

Descriptive parameters of genetic diversity, including the average number of alleles, level of polymorphism (percent of loci that were polymorphic), and observed and expected heterozygosity by sex and by locality were obtained using the GenAlEx software 6.5 package [39]. In some cases, the locality also corresponded directly to specific host plants. A Chi-square test was performed to evaluate whether the genotypic frequencies observed in the samples of males and females by locality fit an expected frequency under the Hardy–Weinberg equilibrium assumption using the GenAlEx software described. Also, to analyze the possibility of mating preferences, a comparison was made of the observed genotypic frequencies and those expected assuming random mating (depending on the allelic frequencies observed in the samples of each sex) [32]. The statistical significance of the comparisons between the observed and expected values within sexes were determined using a χ^2^ test in a spreadsheet from Microsoft Excel version 16.37.

#### 2.4.2. Genetic Diversity by Factors Associated with Host Species and Locality

Diversity parameters (average number of alleles, level of polymorphism, observed and expected heterozygosity) were calculated according to host species without distinction of locality, and to species within locality. We tested whether the observed genotypic frequencies fit the Hardy–Weinberg equilibrium (HWE) model for each locus in a sample arrangement by species, without distinguishing locality, and by species in each locality. All these analyses were performed using the GenAlEx software 6.5 [39]. A pooled test of χ^2^ for each host was performed in each cluster to estimate whether the equilibrium conditions were met for the set of loci [40].

We also carried out a linear regression analysis of expected heterozygosity as a function of elevation using the software R v.3 (R Core Development Team, 2013). Elevation was the only variable where data was available to analyze the relationship of genetic diversity to an environmental factor.

#### 2.4.3. Genetic Structure

Two approaches were used to determine the underlying genetic structure in the samples. The first approach was accomplished through the analysis of molecular variance (AMOVA) to determine the possible hierarchical distribution of genetic variance (1) between localities and between sexes within each locality, (2) between localities and host species, and (3) between host species. Inbreeding coefficients (Φ) were obtained from each AMOVA to assess the level of differentiation of the sample sets involved. These AMOVA analyses were carry out using the GeneAlex software 6.5 [39].

Additionally, we used a Bayesian approach implemented in the Structure program v. 2.3.4 [41] to reveal the structure that underlies the obtained genetic data. This is a grouping method based on a probabilistic model where *K* genetic groups characterized by allelic frequencies are inferred from parameters established by probability for a hypothetical base population constructed based on observed allelic frequencies, assuming Hardy–Weinberg equilibrium in all loci and linkage equilibrium [42]. We based the choice of *K* on the on the ∆*K* method [43], and for this we ran a series of independent runs from *K* = 1 to *K* = 14 (using all individuals) based on 100,000 iterations and following a burn-in period of 100,000 iterations and five repetitions per *K*. We used a model with admixture and correlated allele frequencies. Once the most probable value for *K* was identified by ∆*K,* we averaged the ancestry ratios over the repetition, and the results were plotted in terms of host locality and sex. We also used a procedure by Puechmaille et al. [44] which describes the use of a method to discard spurious clusters in any subpopulation sampled. In addition, we carried out a principal coordinate analysis (PCoA) using a covariance matrix of genetic distance values standardized within the GenAlEx version 6.5 software package [39].

## 3. Results

### 3.1. Genetic Diversity of Males and Females per Locality

A total of 725 individuals were analyzed using six variable enzyme loci. Among the analyzed individuals, we found two alleles for 6PGDH, Got and ME and three alleles for G6PDH, IDH and PGM. The level of polymorphism was generally greater in females than in males. For all sites, the level of polymorphism was between 66% and 100% for females, while in males it ranged between 33% and 83%, except for two localities in which the level of polymorphism was 100% (Table 2).

Variation was also observed in the average number of alleles between males and females in the different localities. Overall, between 1.3 and 2.5 alleles were recorded on average over nine of the localities, and the number of alleles was slightly higher in females than in males (Table 2). The observed heterozygosity (*Ho*) ranged between 0.056 and 0.317 in females, while for males it was between 0.0 and 0.467. In eight localities, the *Ho* was lower in females than in males (Table 2). For both sexes, the expected heterozygosity was always greater than that observed, and in at least seven locations, it was greater in females than in males. The expected heterozygosity (*He*) interval in females ranged between 0.215 and 0.451 and between 0.083 and 0.517 in males (Table 2). All fixation indexes (*f*) were positive, indicating a deficiency of heterozygotes (Table 2).

The results of the χ^2^ tests to assess Hardy–Weinberg equilibrium for each individual locus and for loci combined at each locality are shown in Table 3 (separately for males and females). Different results were obtained for the various localities surveyed. For example, at the Reforma locality, results for individual enzymes showed cases of both significant and nonsignificant departures from expectation in both males and females. For the San Carlos, Toluca and Talquian localities, all of the individual enzyme tests (for both sexes) showed significant departures from expectation. Results from all of the other localities showed significant departures from expectation for all but a small minority of cases. When the results for individual loci were combined, the tests indicated significant departures from expectations at all locations, for both females and males. In addition, some monomorphic loci were observed at all sites sampled here, except the Reforma location.

The frequencies of the observed genotypes were also significantly different from the expected frequencies under the assumption of random mating in each locality for all loci, except for the 6PGDH locus in the samples from Unión de Juárez and Reforma (Table 4).

### 3.2. Genetic Diversity in Factors Associated with Host and Locality

The percent of polymorphic loci was 100% in samples collected from *C. paradisi* and *M. indica.* In samples from *C. sinensis* and *C. edulis,* polymorphisms ranged from 83% to 100% (Table 5). The average number of alleles varied between 2.0 and 2.5 for all samples, with the highest numbers of alleles being recorded in samples of *A. ludens* from *M. indica* fruits. In terms of heterozygosity, the *Ho* values were also highest in the samples collected from *M. indica,* while overall, these values ranged from a low of 0.014 to a high of 0.392. The *Ho* values were also generally lower than the expected values (*He*). These values ranged between 0.337 and 0.528 overall. The highest *He* values were again observed in the collections from *M. indica,* while the lowest were seen in the collections from *C. sinensis* (Table 5). All fixation indexes (*f*) were also positive, indicating a deficiency of heterozygotes. The linear regression of *He* on elevation was negative, but not significant (Regression coefficient R^2^ = 0.028, 1/9 degree free; slope = −1048.7, *Fisher*-statistic = 0.2622; *p* = 0.621).

### 3.3. Genetic Structure

The analysis of molecular variance revealed significant genetic differentiation at several levels, including among localities and host species as well as between sexes and individuals (Table 6). The value among the 22 samples, representing a combination of localities and sexes (Φ _(total)_ = 0.243), was 1.3 times greater than the differentiation observed among 14 sets combining localities and host plants (Φ = 0.178) and almost 1.9 times greater than the differentiation among the set of just the four host plants (Φ = 0.13). The differentiation between sexes was greater than that of localities (Φ _sex(loc)_ = 0.386), as well as that of the value seen for differentiation among localities and host plants (Table 6).

**Figure 2:**
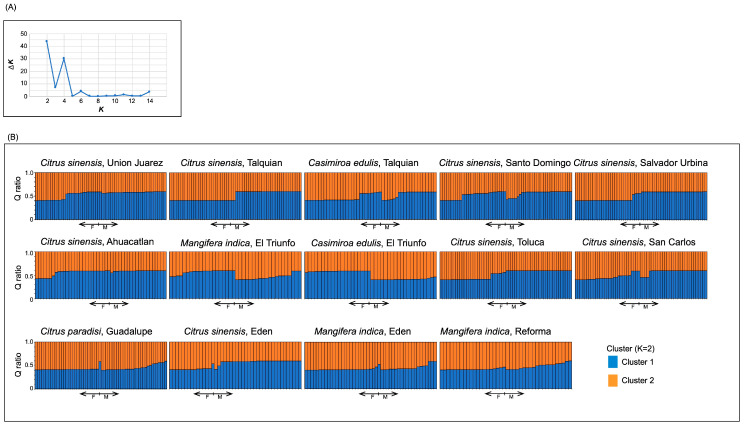
(**A**) ∆*K* calculated following the methods of Evanno et al. [43] and (**B**) Bayesian grouping of samples of *A. ludens* in the Soconusco region based on the Q (membership coefficient matrix) ratio of identified genetic groups. Each bar represents an individual, female (F) or male (M) as indicated, and each color represents the proportion of each genetic cluster. The top label indicates the host and locality of the collection.

**Figure 3:**
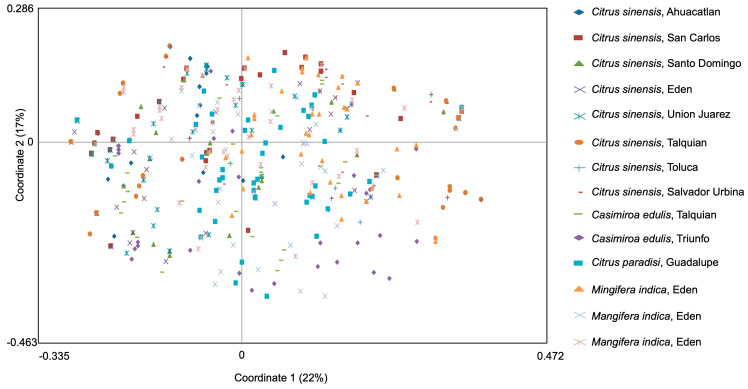
Principal coordinates analysis (PCoA) via covariance matrix with data standardization on genetic distance values.

Using cluster analysis based on ∆*K* methods to detect genetic structuring, possible values for genetic groupings were identified (Figure 2A). However, by graphing individuals in proportions corresponding to these possible groupings according to locality and sex, it can be observed that the composition of each sample does not show significant structuring (Figure 2B). Also, using PCoA, (Figure 3) we found that the first two principal coordinates accounted for 39% of the total variation, and here as well, no obvious clustering is visible.

## 4. Discussion

The results of the present study reveal important new information about genetic variation in populations of *A. ludens* in the Sococusco region of Chiapas, Southern Mexico, including evidence for a lack of structuring of populations in this region due to host plants or geography. Previous work by Dupuis et al. [6], covering a wide geographic range of *A. ludens* populations from Western to Eastern Mexico (including samples from Texas), recognized four broadly defined population groups [6]. In another study, Ruiz-Arce et al. [24] observed genetic differentiation between Mexican populations separated by the Isthmus of Tehuantepec. Our populations were located within this region.

Our study confirmed that overall, moderate to high levels of genetic variation are present in *A. ludens* in the Soconusco region, consistent with results reported by previous studies on this pest species. Our results suggest that the Soconusco population in particular is highly diverse since the overall levels of expected genetic diversity (*He*) found here ranged from 0.337 to 0.528. These values are above those observed in the Mexican populations on a national scale, where values ranging from 0.199 to 0.330 have been reported [23]. Using the same loci as those reported Molina-Neri et al. [23], we found consistently higher levels of heterozygosity (*He* = 0.350). Using other markers such as amplified fragment length polymorphism (AFLP), studies of genetic diversity of *A. ludens* in Northeast Mexico showed *He* values ranging from 0.28 to 0.76 [24,25]. Using double-digest restriction site-associated DNA sequences (ddRAD-Seq), a study considering samples across the range distribution of the species (Texas, Mexico and Central America) produced *He* values between 0.126–0.150 (specifically, for samples from Chiapas, the *He* value was 0.147). In this same study, slightly higher values ranging from 0.162–0.227 were obtained using single nucleotide polymorphisms (SNP) markers [6].

The results we obtained with enzymatic markers might indicate that the Southern populations are more genetically diverse because *A. ludens* in this part of Mexico maintains comparatively large population sizes and is known to have a wider host range with a high degree of dispersal ability [45]. The constant movement of individuals between subpopulations allows the introduction of new alleles that increase the diversity of populations [1], and this can counter the erosive effects of environmental selections either through natural causes or through pest control activities that can substantially reduce population sizes. It is likely that further studies in the Soconusco region using other types of genetic markers based on genomic DNA sequences will show levels of genetic variation close to or higher than those detected with enzyme markers. Thus, more genetic diversity may be uncovered with other molecular markers [46], but this will have to be validated in futures studies.

One of our aims here was to see whether the host plants had an impact on the genetic variation seen for *A. ludens* in the Soconusco region. We recorded the highest level of genetic diversity in the samples associated with mango, while the lowest were observed in the samples from orange (*He* = 0.497 and 0.397, respectively). However, the differences in *He* values were relatively small between the different host species considered here. This may suggest that all these hosts (*C. sinensis, C. paradisi, C. edulis* and *M. indica*), as well as other plant species known to be hosts [4], may represent an environment that supports similar levels of fitness and genetic diversity. Consistent with this, Pecina-Quintero et al. [25] found similar levels of genetic diversity between samples of *A. ludens* obtained from two different hosts, *Casimiroa gregii* and *Citrus sinensis*, in Northeast Mexico using AFLP markers. Genetic differences among hosts were, however, significant in the AMOVA analyses, indicating that host species do have an impact on genetic variation [25]. In our study, a difference of 13% among hosts was detected, and this contrasts sharply with a 3% difference observed between *Casimiroa gregii* and *Citrus sinensis* in Northeast Mexico observed in another study [26]. However, we did not detect significant genetic structuring of the populations based on locality.

Other factors associated with host plants can exert selection, promoting the type of genetic differentiation detected in our AMOVA analyses. These include relative abundance [47], nutritional contributions of the host [48], and the occurrence of endosymbionts of the pests [49,50] that might also impact the use of one host or another. The role of these and other factors in shaping the genetic diversity of *A. ludens* could be addressed in future studies using genetic markers other than enzymatic loci in order to capture more of the genetic variation that might be present here. The use of these other markers could also confirm whether the lack of genetic structuring we saw in subpopulations of *A*. *ludens* in relationship to host plant species in the Soconusco region is real.

Regarding our second aim, we were also interested in the possible impact of other environmental factors because of the significant genetic differences we found among localities. However, the only environmental factor with enough data to study here was elevation. Elevation is associated with variation in temperature, relative humidity and rainfall [51]. Overall, a level of genetic differentiation between localities of 17.8% was observed, but the regression analysis did not show a significant relationship of *He* with elevation. Previous studies, such as that of Molyna-Nery et al. [23], showed that genetic differentiation between geographically distant populations could be attributed to environmental factors such as temperature, but also that interactions with the availability of hosts was always a factor. Pecina-Quintero et al. [25] also reported a relatively high value for Wright’s coefficient of differentiation *F*st (0.38) associated with habitat fragmentation, genetic drift, or local selection factors such as climate and pest management.

Our third aim was to explore whether genetic variation can be associated with the sex of the flies. This was based on the knowledge that this species has a lek–polygamous mating system, characterized by non-random mating [52], and in such cases the direction of mating preferences may have an impact on the overall levels of population genetic diversity [53]. A general trend we found was that females showed higher levels of genetic variation than males. In some locations, it was almost twice as high, and this result is not what would be expected based simply on random mating. In several studies, differences in allele frequencies between males and females have been used to look at the issue of sexual conflict between species [34] In leaf beetles, for example, non-random differences in gene frequencies between females and males, attributed to differences in their dispersal behavior, have been observed [54]. In some birds, in particular those where sexual selection has been well documented, levels of heterozygosity were positively correlated with the survival and fitness of females, but not males [55].

For *A. ludens*, variation in sexual competitiveness has been observed under controlled conditions [56,57,58]. The importance of this is likely to be enhanced in wild populations engaging in leks where females choose the males [59] and some females fail to reproduce due to selection against them [60]. The trend of lower genetic variation (*He*) in males may also reflect a selective balance on alleles that have a positive effect on the fitness of the female but a negative effect in the male [34], [60,61,62]. In *Drosophila*, [63] describes how sexually antagonistic variation may promote traits that enhance the reproductive success of one sex, even at a fitness cost to their mating partners.

Overall, of course, multiple factors can contribute to the maintenance of high levels of genetic variation in any population. It is also important to note that we analyzed these loci as autosomal genes. The possibility of X-linked genes cannot be ruled out with the present data, but this situation has been shown to promote similar allelic frequencies in both sexes if random mating occurs [32]. Finally, phenomena such as meiotic drive and linkage disequilibrium may also impact allele frequencies in populations. Meiotic drive has been seen in some *Drosophila* species [64], but this phenomenon has not been reported for *A. ludens*. Examination of the effects of linkage disequilibrium on the *A. ludens* populations studied here, which is recognized to be important in understanding the landscape of genetic variation in other species [65,66], will be left to future studies incorporating additional loci or genetic markers.

As part of our analysis of the relatively high levels of diversity, we also found genetic differences by locality, host and sex with respect to the HWE model. However, given that *A. ludens* is a species with a high reproductive rate [67], a wide dispersal capacity [68], and random mating (at least among geographically distant populations [21]), the importance of selection may be difficult to distinguish from effects due to drift, recombination, and other factors [32]. It is also true that at each stage of development, *A. ludens* undergoes natural selection that can differ both in intensity, mode, and direction [69], and this may contribute to patterns of genetic variation.

Overall, our results also suggest that sexual selection can be of great importance in the population genetic structure of these flies. The genetic differences seen between the sexes can arise from non-random mating when mates of specific phenotypes of one or both sexes are preferred. The differential ability of the sexes to transmit genes to the next generation is, by definition, sexual selection [70]. The AMOVA analysis showed that the difference between the sexes was relatively high at 38.6% (Φ _sex(loc)_), but when localities and hosts were considered, the differentiation fell to 17.8% (Φ _loc-host_). The genetic differences between males and females may also be due to the way that matings occur. Females are attracted to leks, and it is here that they choose the males to mate with. This could be seen as an example of assortative mating [53]. In addition, females are known to have greater mobility between populations [4,5], and there are also differences in sexual maturation that may reduce the possibility of mating [71]. Furthermore, any selection of host plants may be due to the chemical or nutritional characteristics of the plant species. A demographic study showed that the performance of *A. ludens* larvae in *C. sinensis* and *M. indica* differed significantly and could be attributed to the nutritional contributions of the host plant [22]. In other insect groups, secondary compounds are considered to be a main factor in selection [14].

## 5. Conclusions

We can conclude that the high levels of genetic diversity of *A. ludens* seen in the Soconusco region can be shaped by multiple factors in different localities, including various environmental factors and sexual selection. Of course, factors not included in our study may also play a role. In terms of practical applications, the significant but relatively low levels of differentiation overall among locations, and the lack of clear genetic structuring along with the known sexual compatibility of individuals from populations of different geographic origin [52] support the idea that pest control measures can be used effectively at an area-wide or regional level. However, the potential role of the different plant hosts in any management strategies still needs to be taken into account. In the future, it will also be important to know if the application of the sterile insect technique (SIT) modifies the patterns of male/female genetic diversity and the overall structure of the wild populations. Finally, the genetic differentiation between the sexes observed in the present study suggests the need to place greater attention on behavior and fitness parameters for individuals of both sexes. The application of SIT, for example, could increase the ability of females to selectively recognize wild males and produce more offspring, thus reducing the effectiveness of this control measure [72].

## Figures and Tables

**Table 1:** Collection localities and hosts of *Anastrepha ludens* in the Soconusco region of Chiapas, Mexico. CC, *Citrus sinensis*; CP, *Citrus paradisi*; MI, *Mangifera indica*; CE, *Casimiroa edulis*.

Locality Name	Longitude	Latitude	Elevation (m)	CC	CP	MI	CE
Reforma	−92.32851614	15.0403897	387			x	
Guadalupe	−92.27440112	15.01203335	427		x		
El Triunfo	−92.22400912	14.98588244	470			x	x
San Carlos	−92.26178333	15.04050278	532	x			
Toluca	−92.24566958	15.02878192	538	x			
Salvador Urbina	−92.21133333	15.03722222	540	x			
El Eden	−92.30475556	15.05781389	551	x		x	
Ahuacatlan	−92.17528333	15.042325	719	x			
Santo Domingo	−92.09836944	15.03047222	918	x			
Unión Juárez	−92.07942076	15.06620901	1386	x			
Talquian	−92.08379166	15.08690091	1696	x			x

**Table 2:** Genetic diversity estimators for samples of males and females of *Anastrepha ludens* per locality and for the whole sample (female and male) from 11 localities in the Soconusco region, Chipas, Mexico. N, sample size; Na, average number of alleles; *Ho*, observed heterozygosity; *He*, expected heterozygosity; *f*, fixation index; P, percentage of polymorphic loci. Mi, *Mangifera indica*; Cp, *Citrus paradisi*; Ce, *Casimiroa edulis*; Cc, *Citrus sinensis.*

Locality (Host)	Sex	N	Na	*H*_o_	*H*_e_	*f*	*P*
Reforma (Mi)	Female	20	2.3	0.317	0.451	0.245	100.00
	Male	20	2.0	0.467	0.434	−0.091	100.00
			2.5	0.392	0.528	0.241	100.00
Guadalupe (Cp)	Female	30	1.8	0.056	0.215	0.812	66.67
	Male	30	2.3	0.178	0.468	0.630	100.00
			2.3	0.117	0.410	0.731	100.00
El Triunfo (Ce, Mi)	Female	40	2.2	0.188	0.360	0.452	83.33
	Male	40	2.3	0.329	0.517	0.362	100.00
			2.3	0.258	0.517	0.503	100.00
San Carlos (Cc)	Female	29	2.2	0.241	0.369	0.490	83.33
	Male	30	2.0	0.189	0.299	0.538	83.33
			2.3	0.215	0.447	0.646	100.00
Toluca (Cc)	Female	30	2.0	0.028	0.410	0.933	83.33
	Male	30	1.2	0.000	0.083	1.000	16.67
			2.2	0.014	0.379	0.961	100.00
Salvador Urbina (Cc)	Female	30	2.0	0.089	0.354	0.671	83.33
	Male	30	1.3	0.006	0.148	0.963	33.33
			2.5	0.392	0.528	0.241	83.33
Edén (Cc)	Female	42	2.5	0.190	0.445	0.493	100.00
	Male	49	2.2	0.197	0.413	0.543	83.33
			2.5	0.194	0.484	0.611	100.00
Ahuacatlán (Cc)	Female	27	2.0	0.154	0.241	0.610	66.67
	Male	20	1.8	0.250	0.341	0.302	66.67
			2.2	0.195	0.334	0.566	83.33
Santo Domingo (Cc)	Female	30	2.3	0.117	0.391	0.710	100.00
	Male	30	2.0	0.222	0.368	0.477	83.33
			2.3	0.169	0.417	0.637	100.00
Unión Juárez (Cc)	Female	31	2.2	0.102	0.247	0.643	83.33
	Male	30	1.8	0.106	0.249	0.481	66.67
			2.3	0.104	0.339	0.725	100.00
Talquian (Cc, Ce)	Female	58	2.2	0.075	0.412	0.823	100.00
	Male	50	2.0	0.113	0.331	0.658	83.33
			2.3	0.093	0.488	0.798	100.00
Grand mean over loci and populations (22)	Mean	33	2.0	0.337	0.343	0.545	80.30
	SE		0.0	0.018	0.018	0.038	4.61

**Table 3:** Results of pooled Chi-square analyses of loci to test for Hardy–Weinberg equilibrium in *Anastrepha ludens* male and female samples collected at 11 localities of the Soconusco region in Chiapas, Mexico. The analysis of loci is shown in Table S1. (Supplement 01) *d.f.,* degree of freedom.

Locality	Females			Males		
	χ^2^	*p*	*d.f*.	χ^2^	*p*	*d.f.*
Reforma	38.4	0.0001	12	25.7	0.0120	12
Guadalupe	117.6	<0.0001	10	101.0	0.0000	12
El Triunfo	103.9	<0.0001	10	59.0	<0.0001	12
San Carlos	99.7	<0.0001	8	129.7	<0.0001	10
Toluca	149.0	<0.0001	10	30.0	<0.0001	1
Salvador Urbina	124.3	<0.0001	10	19.1	0.0007	4
Edén	128.8	<0.0001	8	109.9	<0.0001	5
Ahuacatlán	121.1	<0.0001	8	50.1	<0.0001	8
Santo Domingo	128.8	<0.0001	12	89.9	<0.0001	10
Unión Juárez	105.1	<0.0001	8	40.1	<0.0001	10
Talquian	276.9	<0.0001	12	134.5	<0.0001	10

**Table 4:** Results of the χ^2^ analyses to test for random mating of *Anastrepha ludens* collected from 11 localities in the Soconusco region, in Chiapas, Mexico: ^a^, loci with only two alleles; ^b^, loci with three alleles; NS, not significant; M, monomorphic locus; *p* * < 0.05, **; < 0.01, *** < 0.001. Details of the results of this analysis are in Table S2. (Supplement 02) ME, malate dehydrogenase; IDH, isocitrate dehydrogenase; 6PGDH, 6-phosphoglucanate dehydrogenase, G6PDH, glucose-6-phosphate dehydrogenase; GOT, aspartate amino transferase; GPI, glucose-6-ghosphate Isomerase; PGM, Phosphoglucomutase.

Locality	Locus					
	6PGDH ^a^	G6PDH ^b^	GOT ^a^	IDH ^b^	ME ^a^	PGM ^b^
Reforma	3.96 ^NS^	30.37 ***	10.97 *	30.04 ***	16.77 ***	28.34 ***
Guadalupe	20.18 ***	268.97 ***	44.90 ***	46.24 ***	32.49 ***	56.30 ***
El Triunfo	76.51 ***	88.20 ***	50.69 ***	59.01 ***	9.90 ***	472.38 ***
San Carlos	52.60 ***	30.85 ***	38.19 ***	24.50 ***	859.43 ***	39.77 ***
Toluca	103.56 ***	1296.00 ***	59.84 ***	233.68 ***	M	317.04 ***
Salvador Urbina	344.20 ***	2649.05 ***	50.44 ***	76.92 ***	M	126.56 ***
Edén	27.02 ***	61.30 ***	60.67 ***	44.04 ***	64.60 ***	287.42 ***
Ahuacatlán	25.30 ***	15.91 ***	39.49 ***	34.05 ***	161.87 ***	M
Santo Domingo	29.05 ***	22.40 ***	55.19 ***	16.84 **	80.97 ***	50.32 ***
Unión Juárez	1.00 ^NS^	9.38 *	44.11 ***	69.07 ***	595.12 ***	147.80 ***
Talquian	268.75 ***	4071.77 ***	89.56 ***	44.28 ***	97.75 ***	671.46 ***

**Table 5:** Genetic diversity of *Anastrepha ludens* by host species and locality in the Soconusco region, Chiapas (Mexico). N, sample size; Na, average number of alleles; *H_o_*, observed heterozygosity; *H_e_*, unbiased heterozygosity; *P*, percentage of polymorphism; *f* fixation index as *f* = 1 − (*H*_o_/*H*_e_).

Host	Locality	N	*P*	Na	*H*_o_	*H*_e_	*f*	Mean					
								*P*	N	Na	*H*_o_	*H*_e_	*f*
*Citrus sinensis*	San Carlos	59	100.0	2.333	0.215	0.447	0.646	91.67	56	2.271	0.118	0.397	0.744
	Toluca	60	83.33	2.167	0.014	0.379	0.961						
	Salvador Urbina	60	83.33	2.167	0.047	0.381	0.896						
	Edén	44	83.33	2.167	0.110	0.409	0.740						
	Ahuacatlán	47	83.33	2.333	0.191	0.337	0.580						
	Santo Domingo	60	100.0	2.333	0.169	0.417	0.637						
	Unión Juárez	61	100.0	2.333	0.104	0.339	0.725						
	Talquián	60	100.0	2.333	0.094	0.470	0.769						
*Casimiroa edulis*	El Triunfo	40	100.0	2.167	0.171	0.447	0.599	91.67	49	2.167	0.126	0.414	0.697
	Talquián	48	83.33	2.000	0.090	0.386	0.761						
*Citrus paradisi*	Guadalupe	60	100.0	2.333	0.117	0.410	0.731						
*Mangifera indica*	Reforma	40	100.0	2.500	0.392	0.528	0.241	100.0	42	2.444	0.337	0.497	0.911
	El Triunfo	40	100.0	2.333	0.346	0.491	0.296						
	Edén	47	100.0	2.500	0.273	0.474	0.366						
**Mean over 14 samples**								94.05	52	2.286	0.167	0.422	0.301

**Table 6:** Analysis of molecular variance for different hierarchical arrangements of samples of *A. ludens* obtained from 11 localities in the Soconusco region in Chiapas, Mexico. Φ _loc_, level of differentiation among localities; Φ _sex(loc),_ differentiation between sex in relation to locality; Φ _(total),_ differentiation among all samples (22).

Hierarchical Arrangement	Source of Variation	d.f.	Sum of Squares	Medium Square	Estimated Variance	Variance Component (%)	Differentiation Estimation
Locality, sex	Among localities	10	482.93	48.29	0.00	0	Φ _loc_ = 0.0 Φ _sex(loc)_ = 0.386 * Φ _(total)_ = 0.243 *
	Between sexes (locality)	11	784.20	71.29	2.067	39	
	Within individuals	704	2318.50	3.29	3.29	61	
	Total	725	3585.63		5.4	100	
Localities	Among localities	14	653.902	50.3	0.892	18	Φ = 0.178 ***
	Within individuals	712	2931.73	4.12	4.12	82	
	Total	725	3585.63		5.01	100	
Hosts	Among hosts	3	292.4	97.47	0.683	13	Φ = 0.13 ***
	Within individuals	722	3293.2	4.56	4.561	87	
	Total	725	3585.6		5.244	100	

*, *p* = 0.01; ***, *p* < 0.0001.

## Supplementary Materials

The following are available online at https://www.mdpi.com/2075-4450/11/11/815/s1. Table S1. Results of the **χ^2^** analysis for Hardy-Weinberg equilibrium by individual loci and over all loci in *Anastrepha ludens* male and female samples collected at 11 localities of the Soconusco region in Chiapas, México. (Supplement 01) Table S2. Allele frequencies of six locus in samples of males and females of *Anastrepha ludens* collected from 11 localities in the Soconusco region, in Chiapas, Mexico. (Supplement 02).
