# Supplementary material for: Genetic and Ecological Relationships of Anastrepha ludens (Diptera: Tephritidae) Populations in Southern Mexico
Source: Insects. 2020 Nov 19;11(11):815. doi: 10.3390/insects11110815 (PMC7699260; doi:10.3390/insects11110815)
Supplement: Supplementary file 1 [file insects-11-00815-s001.zip › Supplement 02 final.docx]

**Sup**p**lement 02**. Allele frequencies of six locus in samples of males and females of *Anastrepha ludens* collected from 11 localities in the Soconusco region, in Chiapas, Mexico. Genotypic frequencies observed in the whole of the sample by locality, and **χ^2^** test between the observed and expected genotypic frequencies under an assumption of random mating. ^a^, loci with only two alleles; ^b^, loci with three alleles, NS, not significant; M, monomorphic locus; * <0.05, **; <0.01, *** <0.001.

|  |  | Females | | | Males | | | Genotypes | | | |  |
| --- | --- | --- | --- | --- | --- | --- | --- | --- | --- | --- | --- | --- |
| **Locus** | **Locality** | **alles** | | | **alleles** | | | ***P*** | ***H*** | **Q** | **R** | **χ^2^** |
|  |  | ***p*** | ***q*** | ***r*** | ***p*** | ***q*** | ***r*** |  |  |  |  |  |
| 6PGDH^a^ | Reforma | 0.700 | 0.300 |  | 0.375 | 0.625 |  | 12 | 10 | 9 |  | 3.96^NS^ |
|  | Guadalupe | 1.000 | 0.000 |  | 0.833 | 0.167 |  | 54 | 0 | 4 |  | 20.18*** |
|  | El Triunfo | 0.913 | 0.088 |  | 0.475 | 0.525 |  | 49 | 3 | 18 |  | 76.51*** |
|  | San Carlos | 0.276 | 0.724 |  | 0.867 | 0.133 |  | 30 | 8 | 21 |  | 52.60*** |
|  | Toluca | 0.633 | 0.367 |  | 1.000 | 0.000 |  | 49 | 0 | 11 |  | 103.56*** |
|  | Salvador Urbina | 0.317 | 0.683 |  | 1.000 | 0.000 |  | 38 | 3 | 19 |  | 344.20*** |
|  | Edén | 0.500 | 0.500 |  | 0.653 | 0.347 |  | 47 | 8 | 32 |  | 27.02*** |
|  | Ahuacatlán | 0.759 | 0.241 |  | 1.000 | 0.000 |  | 40 | 1 | 6 |  | 25.30*** |
|  | Santo Domingo | 0.800 | 0.200 |  | 0.800 | 0.200 |  | 47 | 0 | 11 |  | 29.05*** |
|  | Unión Juárez | 0.968 | 0.032 |  | 1.000 | 0.000 |  | 60 | 0 | 1 |  | 1.00^NS^ |
|  | Talquian | 0.138 | 0.862 |  | 0.900 | 0.100 |  | 72 | 4 | 30 |  | 268.75*** |
|  |  |  |  |  |  |  |  |  |  |  |  |  |
| G6PDH^b^ | Reforma | 0.075 | 0.925 | 0.000 | 0.300 | 0.300 | 0.400 | 0 | 3 | 20 | 1 | 30.37*** |
|  | Guadalupe | 1.000 | 0.000 | 0.000 | 0.600 | 0.283 | 0.000 | 31 | 0 | 16 | 6 | 268.97*** |
|  | El Triunfo | 0.525 | 0.138 | 0.338 | 0.213 | 0.550 | 0.238 | 24 | 7 | 17 | 15 | 88.20*** |
|  | San Carlos | 0.190 | 0.448 | 0.362 | 0.333 | 0.167 | 0.500 | 0 | 17 | 10 | 2 | 30.85*** |
|  | Toluca | 0.467 | 0.533 | 0.000 | 0.000 | 0.000 | 1.000 | 14 | 0 | 16 | 30 | 1296.00*** |
|  | Salvador Urbina | 0.867 | 0.133 | 0.000 | 0.000 | 0.000 | 1.000 | 52 | 8 | 0 | 0 | 2649.05*** |
|  | Edén | 0.071 | 0.810 | 0.119 | 0.224 | 0.306 | 0.469 | 2 | 10 | 38 | 13 | 61.30*** |
|  | Ahuacatlán | 0.185 | 0.296 | 0.519 | 0.450 | 0.100 | 0.450 | 0 | 24 | 1 | 2 | 15.91*** |
|  | Santo Domingo | 0.150 | 0.717 | 0.133 | 0.183 | 0.400 | 0.417 | 1 | 11 | 20 | 2 | 22.40*** |
|  | Unión Juárez | 0.048 | 0.806 | 0.145 | 0.050 | 0.800 | 0.150 | 0 | 10 | 41 | 0 | 9.38* |
|  | Talquian | 0.336 | 0.664 | 0.000 | 0.400 | 0.000 | 0.600 | 35 | 9 | 64 | 0 | 4071.77*** |
|  |  |  |  |  |  |  |  |  |  |  |  |  |
| GOT^a^ | Reforma | 0.500 | 0.500 |  | 0.200 | 0.800 |  | 7 | 8 | 23 | 0 | 10.97* |
|  | Guadalupe | 0.117 | 0.883 |  | 0.367 | 0.633 |  | 12 | 1 | 43 | 0 | 44.90*** |
|  | El Triunfo | 0.250 | 0.750 |  | 0.500 | 0.500 |  | 24 | 6 | 44 | 0 | 50.69*** |
|  | San Carlos | 0.103 | 0.897 |  | 0.200 | 0.800 |  | 9 | 0 | 50 | 0 | 38.19*** |
|  | Toluca | 0.633 | 0.367 |  | 0.433 | 0.567 |  | 32 | 0 | 28 | 0 | 59.84*** |
|  | Salvador Urbina | 0.200 | 0.800 |  | 0.300 | 0.700 |  | 15 | 0 | 45 | 0 | 50.44*** |
|  | Eden | 0.476 | 0.524 |  | 0.306 | 0.694 |  | 29 | 4 | 50 | 0 | 60.67*** |
|  | Ahuacatlán | 0.037 | 0.963 |  | 0.425 | 0.575 |  | 7 | 0 | 35 | 0 | 39.49*** |
|  | Santo Domingo | 0.583 | 0.417 |  | 0.267 | 0.733 |  | 23 | 1 | 32 | 0 | 55.19*** |
|  | Unión Juárez | 0.081 | 0.919 |  | 0.283 | 0.717 |  | 10 | 1 | 49 | 0 | 44.11*** |
|  | Talquian | 0.586 | 0.414 |  | 0.330 | 0.670 |  | 44 | 2 | 51 | 0 | 89.56*** |
|  |  |  |  |  |  |  |  |  |  |  |  |  |
| IDH^b^ | Reforma | 0.675 | 0.300 | 0.025 | 0.250 | 0.600 | 0.150 | 13 | 2 | 13 | 2 | 30.04*** |
|  | Guadalupe | 0.533 | 0.433 | 0.033 | 0.233 | 0.617 | 0.150 | 18 | 6 | 25 | 4 | 46.24*** |
|  | El Triunfo | 0.100 | 0.438 | 0.463 | 0.275 | 0.600 | 0.125 | 6 | 9 | 26 | 16 | 59.01*** |
|  | San Carlos | 0.345 | 0.466 | 0.190 | 0.600 | 0.400 | 0.000 | 20 | 17 | 16 | 4 | 24.50*** |
|  | Toluca | 0.583 | 0.400 | 0.017 | 0.000 | 1.000 | 0.000 | 15 | 5 | 40 | 0 | 233.68*** |
|  | Salvador Urbina | 0.450 | 0.333 | 0.217 | 0.350 | 0.650 | 0.000 | 21 | 5 | 27 | 6 | 76.92*** |
|  | Eden | 0.214 | 0.714 | 0.071 | 0.296 | 0.684 | 0.020 | 14 | 4 | 54 | 2 | 44.04*** |
|  | Ahuacatlán | 0.111 | 0.778 | 0.111 | 0.325 | 0.675 | 0.000 | 7 | 0 | 32 | 3 | 34.05*** |
|  | Santo Domingo | 0.167 | 0.800 | 0.033 | 0.250 | 0.750 | 0.000 | 7 | 6 | 40 | 0 | 16.84** |
|  | Unión Juárez | 0.113 | 0.726 | 0.161 | 0.533 | 0.467 | 0.000 | 17 | 6 | 33 | 3 | 69.07*** |
|  | Talquian | 0.172 | 0.776 | 0.052 | 0.250 | 0.740 | 0.010 | 15 | 9 | 73 | 0 | 44.28*** |
|  |  |  |  |  |  |  |  |  |  |  |  |  |
| ME^a^ | Reforma | 0.450 | 0.550 |  | 0.325 | 0.675 |  | 8 | 2 | 17 | 0 | 16.77*** |
|  | Guadalupe | 0.383 | 0.617 |  | 0.450 | 0.550 |  | 19 | 3 | 29 | 0 | 32.49*** |
|  | El Triunfo | 0.300 | 0.700 |  | 0.425 | 0.575 |  | 14 | 20 | 36 | 0 | 9.90*** |
|  | San Carlos | 1.000 | 0.000 |  | 0.067 | 0.933 |  | 30 | 0 | 27 | 0 | 859.43*** |
|  | Toluca | 1.000 | 0.000 |  | 1.000 | 0.000 |  | 60 | 0 | 0 | 0 | M |
|  | Salvador Urbina | 1.000 | 0.000 |  | 1.000 | 0.000 |  | 60 | 0 | 0 | 0 | M |
|  | Eden | 0.833 | 0.167 |  | 0.439 | 0.561 |  | 49 | 14 | 27 | 0 | 64.60*** |
|  | Ahuacatlán | 1.000 | 0.000 |  | 0.400 | 0.600 |  | 35 | 0 | 12 | 0 | 161.87*** |
|  | Santo Domingo | 0.800 | 0.200 |  | 0.350 | 0.650 |  | 34 | 0 | 25 | 0 | 80.97*** |
|  | Unión Juárez | 1.000 | 0.000 |  | 0.133 | 0.867 |  | 32 | 0 | 23 | 0 | 595.12*** |
|  | Talquian | 0.707 | 0.293 |  | 0.380 | 0.620 |  | 54 | 2 | 42 | 0 | 97.75*** |
|  |  |  |  |  |  |  |  |  |  |  |  |  |
| PGM^b^ | Reforma | 0.200 | 0.475 | 0.325 | 0.950 | 0.050 | 0.000 | 20 | 13 | 3 | 2 | 28.34*** |
|  | Guadalupe | 0.033 | 0.967 | 0.000 | 0.317 | 0.683 | 0.000 | 9 | 0 | 48 | 0 | 56.30*** |
|  | El Triunfo | 1.000 | 0.000 | 0.000 | 0.313 | 0.688 | 0.000 | 46 | 0 | 21 | 0 | 472.38*** |
|  | San Carlos | 0.793 | 0.207 | 0.000 | 1.000 | 0.000 | 0.000 | 53 | 0 | 6 | 0 | 39.77*** |
|  | Toluca | 0.433 | 0.567 | 0.000 | 1.000 | 0.000 | 0.000 | 53 | 0 | 17 | 0 | 317.04*** |
|  | Salvador Urbina | 0.633 | 0.367 | 0.000 | 1.000 | 0.000 | 0.000 | 49 | 0 | 11 | 0 | 126.56*** |
|  | Eden | 0.405 | 0.476 | 0.119 | 1.000 | 0.000 | 0.000 | 64 | 8 | 16 | 3 | 287.42*** |
|  | Ahuacatlán | 1.000 | 0.000 | 0.000 | 1.000 | 0.000 | 0.000 | 47 | 0 | 0 | 0 | M |
|  | Santo Domingo | 0.717 | 0.283 | 0.000 | 1.000 | 0.000 | 0.000 | 50 | 3 | 7 | 0 | 50.32*** |
|  | Unión Juárez | 0.581 | 0.419 | 0.000 | 1.000 | 0.000 | 0.000 | 47 | 2 | 12 | 0 | 147.80*** |
|  | Talquian | 0.517 | 0.483 | 0.000 | 1.000 | 0.000 | 0.000 | 58 | 4 | 26 | 0 | 671.46*** |
