# Supplementary material for: Genetic and Ecological Relationships of Anastrepha ludens (Diptera: Tephritidae) Populations in Southern Mexico
Source: Insects. 2020 Nov 19;11(11):815. doi: 10.3390/insects11110815 (PMC7699260; doi:10.3390/insects11110815)
Supplement: Supplementary file 1 [file insects-11-00815-s001.zip › Supplement 01 final.docx]

**Supplement 01.** Results of the **χ^2^** analysis for Hardy-Weinberg equilibrium by individual loci and over all loci in *Anastrepha ludens* male and female samples collected at 11 localities of the Soconusco region in Chiapas, México. Mi, *Manguifera indica*; Cp, *Citrus paradisi*; Ce, *Casimeroa edulis*; Cc, *Citrus sinensis*

| Locality | Locus | Females | | |  | Males | | |
| --- | --- | --- | --- | --- | --- | --- | --- | --- |
|  |  | DF | **χ^2^** | *P* |  | DF | **χ^2^** | *P* |
| Reforma | 6PGDH | 1 | 0.7 | 0.3943 |  | 1 | 0.03 | 0.8580 |
|  | IDH | 3 | 15.9 | 0.0012 |  | 3 | 11.1 | 0.0111 |
|  | ME | 1 | 12.7 | 0.0004 |  | 1 | 4.6 | 0.0313 |
|  | G6PDH | 1 | 0.1 | 0.7169 |  | 3 | 10.6 | 0.0142 |
|  | PGM | 3 | 6.2 | 0.1016 |  | 1 | 0.1 | 0.8139 |
|  | GOT | 1 | 0.8 | 0.3711 |  | 1 | 0.1 | 0.7799 |
|  | Overall |  | 38.4 | 0.0001 |  |  | 25.7 | 0.0120 |
|  |  |  |  |  |  |  |  |  |
| Guadalupe | 6PGDH | Mono |  |  |  | 1 | 17.3 | <0.0001 |
|  | IDH | 3 | 40.2 | <0.0001 |  | 3 | 21.8 | 0.0001 |
|  | ME | 1 | 18.7 | <0.0001 |  | 1 | 4.7 | 0.0310 |
|  | G6PDH | Mono |  |  |  | 3 | 17.0 | 0.0007 |
|  | PGM | 1 | 30.0 | <0.0001 |  | 1 | 17.7 | <0.0001 |
|  | GOT | 1 | 21.1 | <0.0001 |  | 1 | 15.2 | 0.0001 |
|  | Overall |  | 117.6 | <0.0001 |  |  | 101.0 | 0.0000 |
|  |  |  |  |  |  |  |  |  |
| El Triunfo | 6PGDH | 1 | 11.3 | <0.0001 |  | 1 | 9.9 | 0.0016 |
|  | IDH | 3 | 35.0 | <0.0001 |  | 3 | 1.9 | 0.5962 |
|  | ME | 1 | 1.5 | 0.228 |  | 1 | 9.5 | 0.0020 |
|  | G6PDH | 3 | 40.4 | <0.0001 |  | 3 | 7.2 | 0.0665 |
|  | PGM | Mono |  |  |  | 1 | 2.4 | 0.1233 |
|  | GOT | 1 | 14.4 | <0.0001 |  | 1 | 19.6 | <0.0001 |
|  | Overall |  | 103.9 | <0.0001 |  |  | 59.0 | <0.0001 |
|  |  |  |  |  |  |  |  |  |
| San Carlos | 6PGDH | 1 | 2.8 | <0.0001 |  | 1 | 30.0 | <0.0001 |
|  | IDH | 3 | 15.7 | <0.0001 |  | 1 | 22.2 | <0.0001 |
|  | ME | Mono |  |  |  | 1 | 6.5 | 0.0110 |
|  | G6PDH | 3 | 23.4 | <0.0001 |  | 3 | 30.0 | <0.0001 |
|  | PGM | 1 | 29.0 | <0.0001 |  | Mono |  |  |
|  | GOT | 1 | 29.0 | <0.0001 |  | 1 | 30.0 | <0.0001 |
|  | Overall |  | 99.7 | <0.0001 |  |  | 129.7 | <0.0001 |
|  |  |  |  |  |  |  |  |  |
| Toluca | 6PGDH | 1 | 30.0 | <0.0001 |  | Mono |  |  |
|  | IDH | 3 | 15.7 | 0.0012 |  | Mono |  |  |
|  | ME | Mono |  |  |  | Mono |  |  |
|  | G6PDH | 1 | 30.0 | <0.0001 |  | Mono |  |  |
|  | PGM | 1 | 30.0 | <0.0001 |  | Mono |  |  |
|  | GOT | 1 | 30.0 | <0.0001 |  | 1 | 30.0 | <0.0001 |
|  | Overall |  | 149.0 | <0.0001 |  |  |  |  |
|  |  |  |  |  |  |  |  |  |
| Salvador Urbina | 6PGDH | 1 | 17.7 | <0.0001 |  | Mono |  |  |
|  | IDH | 3 | 36.6 | <0.0001 |  | 1 | 25.8 | <0.0001 |
|  | ME | Mono |  |  |  | Mono |  |  |
|  | G6PDH | 1 | 0.7 | 0.399 |  | Mono |  |  |
|  | PGM | 1 | 30.0 | <0.0001 |  | Mono |  |  |
|  | GOT | 1 | 30.0 | <0.0001 |  | 1 | 30.0 | <0.0001 |
|  | Overall |  | 124.3 | <0.0001 |  |  | 19.1 | 0.0007 |
|  |  |  |  |  |  |  |  |  |
| Edén | 6PGDH | 1 | 16.1 | <0.0001 |  | 1 | 32.9 | <0.0001 |
|  | IDH | 3 | 47.7 | <0.0001 |  | 3 | 7.0 | 0.0715 |
|  | ME | 1 | 1.7 | <0.0001 |  | 1 | 45.0 | <0.0001 |
|  | G6PDH | 3 | 7.7 | <0.0001 |  | 3 | 12.0 | 0.0075 |
|  | PGM | 3 | 37.0 | <0.0001 |  | Mono |  |  |
|  | GOT | 1 | 27.5 | <0.0001 |  | 1 | 18.6 | <0.0001 |
|  | Overall |  | 128.8 | <0.0001 |  |  | 109.9 | <0.0001 |
|  |  |  |  |  |  |  |  |  |
| Ahuacatlan | 6PGDH | 1 | 21.8 | <0.0001 |  | Mono |  |  |
|  | IDH | 3 | 54.0 | <0.0001 |  | 1 | 3.7 | 0.0544 |
|  | ME | Mono |  | <0.0001 |  | 1 | 20.0 | <0.0001 |
|  | G6PDH | 3 | 16.9 | 0.001 |  | 3 | 16.0 | 0.0011 |
|  | PGM | Mono |  | <0.0001 |  | Mono |  |  |
|  | GOT | 1 | 27.0 | <0.0001 |  | 1 | 4.8 | 0.0289 |
|  | Overall |  | 121.1 | <0.0001 |  |  | 50.1 | <0.0001 |
|  |  |  |  |  |  |  |  |  |
| Santo Domingo | 6PGDH | 1 | 30.0 | <0.0001 |  | 1 | 18.8 | <0.0001 |
|  | IDH | 3 | 8.3 | 0.041 |  | 1 | 4.3 | 0.0385 |
|  | ME | 1 | 30.0 | <0.0001 |  | 1 | 25.8 | <0.0001 |
|  | G6PDH | 3 | 6.0 | 0.112 |  | 3 | 17.9 | 0.0005 |
|  | PGM | 1 | 17.0 | <0.0001 |  | Mono |  |  |
|  | GOT | 1 | 26.0 | <0.0001 |  | 1 | 13.0 | 0.0003 |
|  | Overall |  | 128.8 | <0.0001 |  |  | 89.9 | <0.0001 |
|  |  |  |  |  |  |  |  |  |
| Unión Juárez | 6PGDH | 1 | 31.0 | <0.0001 |  | Mono |  |  |
|  | IDH | 3 | 17.7 | <0.0001 |  | 1 | 22.5 | 0.4609 |
|  | ME | Mono |  | <0.0001 |  | 1 | 0.5 | 0.0084 |
|  | G6PDH | 3 | 7.2 | 0.065 |  | 3 | 6.9 | 1.0000 |
|  | PGM | 1 | 23.3 | <0.0001 |  | Mono |  |  |
|  | GOT | 1 | 19.0 | <0.0001 |  | 1 | 25.3 | <0.0001 |
|  | Overall |  | 105.1 | <0.0001 |  |  | 40.1 | <0.0001 |
|  |  |  |  |  |  |  |  |  |
| Talquian | 6PGDH | 1 | 58.0 | <0.0001 |  | 1 | 30.2 | <0.0001 |
|  | IDH | 3 | 34.2 | <0.0001 |  | 3 | 12.5 | 0.0059 |
|  | ME | 1 | 48.7 | <0.0001 |  | 1 | 16.6 | <0.0001 |
|  | G6PDH | 1 | 24.7 | <0.0001 |  | 1 | 50.0 | <0.0001 |
|  | PGM | 1 | 43.1 | <0.0001 |  | Mono |  |  |
|  | GOT | 1 | 50.0 | <0.0001 |  | 1 | 12.6 | 0.0004 |
|  | Overall |  | 276.9 | <0.0001 |  |  | 134.5 | <0.0001 |
